# Supplementary material for: Genetic Ablation of the Nutrient Sensor Ogt in Endocrine Progenitors Is Dispensable for β-Cell Development but Essential for Maintenance of β-Cell Mass
Source: Biomedicines. 2022 Dec 30;11(1):105. doi: 10.3390/biomedicines11010105 (PMC9855876; doi:10.3390/biomedicines11010105)
Supplement: Supplementary file 1 [file biomedicines-11-00105-s001.zip › biomedicines-2118626-SI.pdf]

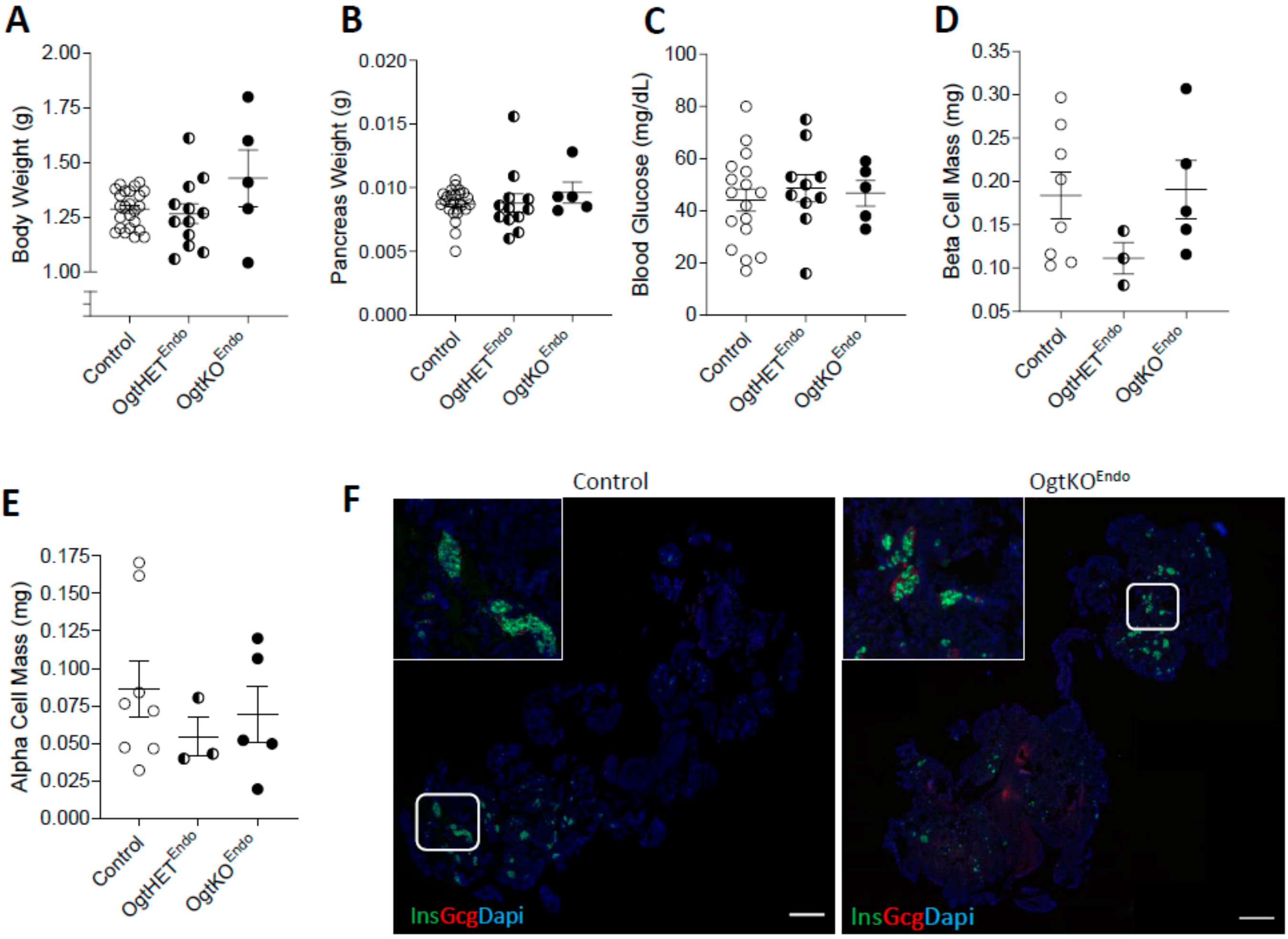

Supplemental Figure S1. Both OgtHET^{Endo} and OgtKO^{Endo} are normal at birth. Body weight (**A**, *n* = 5–24), pancreas weight (**B**, *n* = 5–24), non-fasting blood glucose (**C**, *n* = 5–17), β-cell mass (**D**, *n* = 3–8), and α-cell mass (**E**, *n* = 3–8) at p0. Representative image showing normal endocrine and exocrine pancreas morphology at p0 (**F**, 10×, scale bar = 100 μm). For all graphs, open circle = Control, closed circle = OgtKO^{Endo}, and half-filled circle = OgtHET^{Endo}. Both male and female data combined.

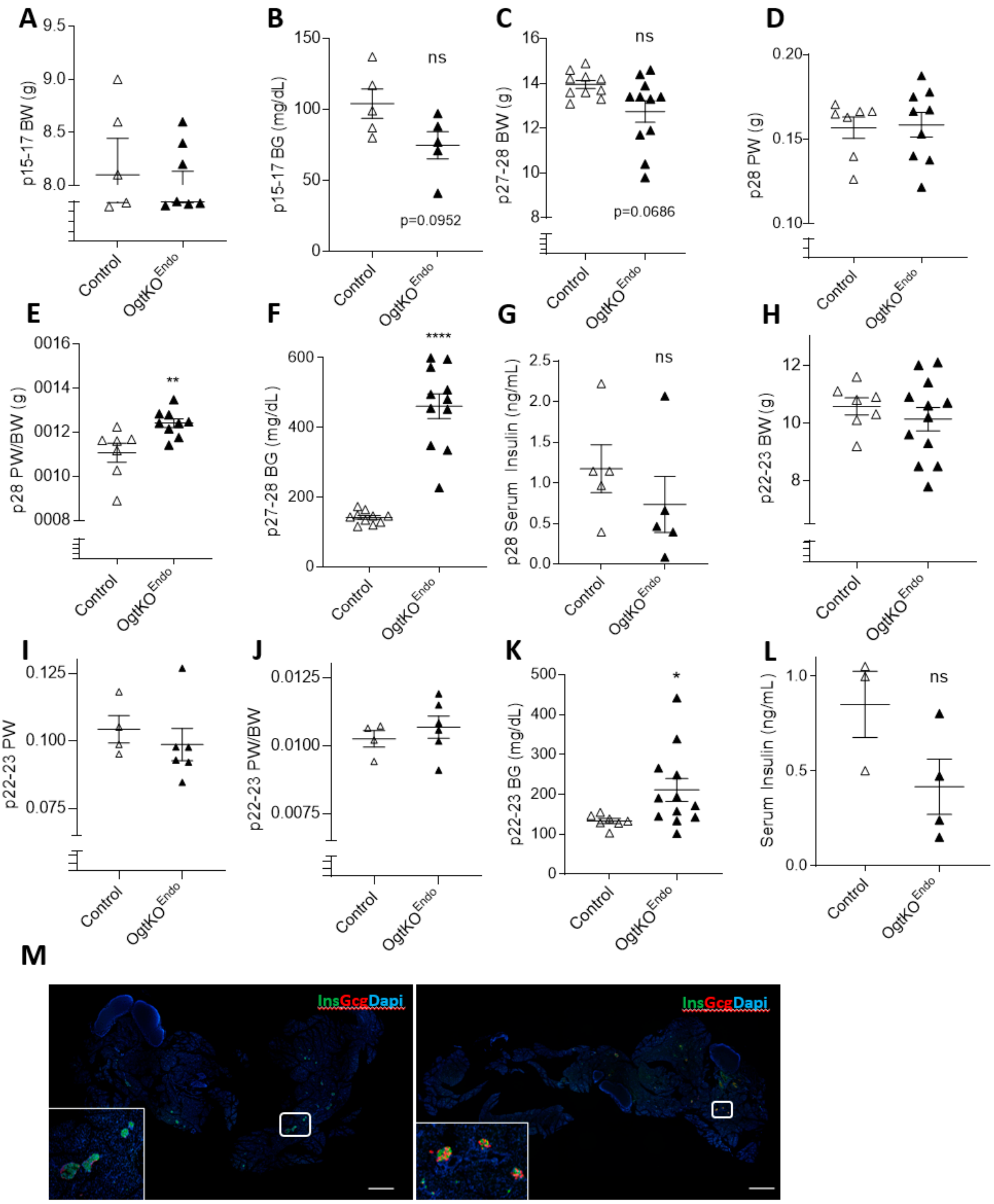

Supplemental Figure S2. Female OgtKO^{Endo} develop progressive hyperglycemia over time. Body weight (**A**, *n* = 5–7) and non-fasting blood glucose (**B**, *n* = 5, *p* = 0.0952) at p15–17. Body weight (**C**, *n* = 10–11, *p* = 0.0686), pancreas weight (**D**, *n* = 7–9), pancreas/body weight (**E**, *n* = 7–9, **p = 0.0052), non-fasting blood glucose (**F**, *n* = 10–11, ****p < 0.0001), and serum insulin (**G**, *n* = 5) at p27-28. Body weight (**H**, *n* = 7–12), pancreas weight (**I**, *n* = 4–6), pancreas/body weight (**J**, n = 4–6), non-fasting blood glucose (**K**, *n* = 7–12, *p = 0.0171), and serum insulin (**L**, *n* = 3–4) and representative images of pancreas morphology (**M**, 4×, scale bar = 1000 μm) at p23. In all graphs, open triangle = Control and closed triangle = OgtKO^{Endo}.

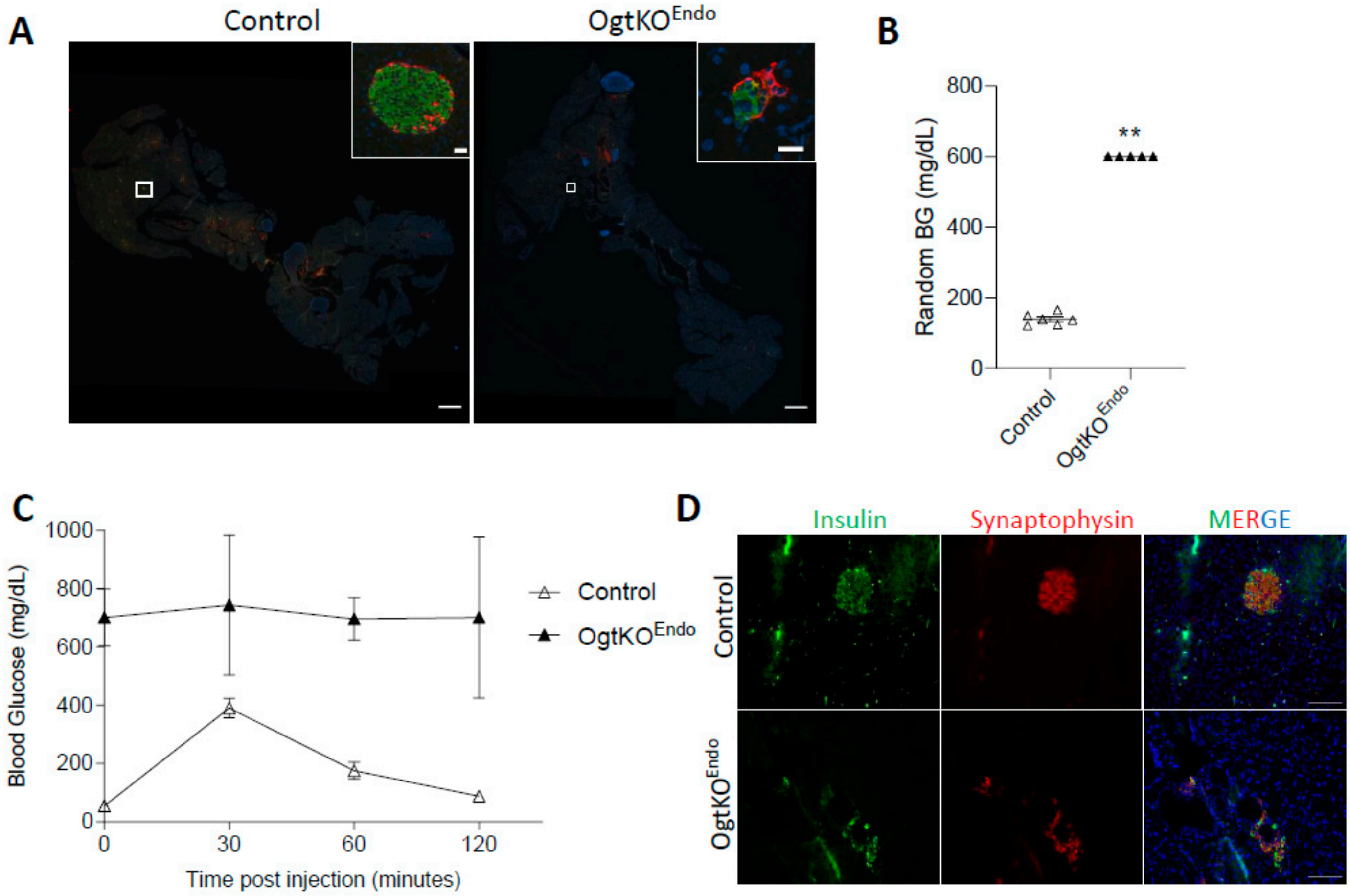

Supplemental Figure S3. Loss of α-cell mass and β-cell mass in OgtKO^{Endo} is not compensated by other pancreatic endocrine cells. Representative immunofluorescence images of whole pancreas at p60 (**A**, 10×, scale = 1000 μm). Non-fasting blood glucose in females (**B**, *n* = 5–6) at p60. Intraperitoneal glucose tolerance test in females (**C**, *n* = 3–6) at p70. Representative images of islets stained with synaptophysin showing no compensation from other islet cells in the OgtKO^{Endo} (**D**, 20×, scale = 50 μm). In all graphs, open triangle = Control and closed triangle = OgtKO^{Endo}. **p≤0.01.
